# Clinical evaluation of tissue-dependent and spatially-variant positron range correction for Gallium-68 PET imaging

**DOI:** 10.1007/s00259-025-07456-z

**Published:** 2025-07-28

**Authors:** Prodromos Gavriilidis, Michel Koole, Felix M. Mottaghy, Floris P. Jansen, Roel Wierts

**Affiliations:** 1 Department of Radiology and Nuclear Medicine, Maastricht University Medical Center, Maastricht, The Netherlands; 2 Research Institute for Oncology and Reproduction (GROW), Maastricht University, Maastricht, The Netherlands; 3 Nuclear Medicine and Molecular Imaging, Department of Imaging & Pathology, KU Leuven, Leuven, Belgium; 4 Department of Nuclear Medicine, RWTH Aachen University Hospital, Aachen, Germany; 5 Molecular Imaging, GE HealthCare, Waukesha, WI USA

**Keywords:** Positron emission tomography, Gallium-68, Positron range, Positron range correction

## Abstract

**Purpose:**

Positron range correction (PRC) can mitigate the effect of the larger positron range on the image quality of Gallium-68 (^68^Ga) PET-imaging. The aim of this study is to evaluate the improvement in ^68^Ga-PET image quality by applying a tissue-dependent and spatially-variant PRC (TDSV PRC) for ^68^Ga in a clinical setting.

**Methods:**

A TDSV PRC technique was developed employing CT-driven segmentation masks of different tissue types (soft tissue, bone, lung) and the corresponding tissue-specific positron range kernels. OSEM reconstructions were performed using the proposed TDSV PRC, a tissue-independent PRC, and without any PRC (non-PRC). For lesions identified in [^68^Ga]Ga-DOTATOC or [^68^Ga]Ga-PSMA PET/CT data from 20 patients, the maximum standardized uptake value (SUV_max_) and contrast-to-noise ratio (CNR) of this technique was compared to tissue-independent PRC and non-PRC images.

**Results:**

A total of 93 lesions were analyzed (48 soft tissue, 35 bone, 10 lung lesions). For soft tissue lesions, TDSV and tissue-independent PRC showed similar increases in SUV_max_ (13.7%, *p* < 0.001 vs. 13.6%, *p* < 0.001) and CNR (11.0%, *p* < 0.001 vs. 11.1%, *p* < 0.001) compared to non-PRC. For bone lesions, tissue-independent PRC showed slightly higher not statistically significant increases than TDSV PRC in SUV_max_ (18.6%, *p* < 0.001 vs. 17.4%, *p* < 0.001) and CNR (14.6%, *p* < 0.001 vs. 13.8%, *p* < 0.001). In lung lesions, TDSV PRC increased SUV_max_ and CNR compared to non-PRC (SUV_max_: 57.9%, *p* = 0.012; CNR: 43.9%, *p* = 0.012) and tissue-independent PRC (SUV_max_: 46.0%, *p* = 0.012; CNR: 32.5%, *p* = 0.012).

**Conclusion:**

TDSV PRC for ^68^Ga PET/CT demonstrated to be feasible in clinical patient data, showing the greatest benefits for lung lesions.

**Supplementary Information:**

The online version contains supplementary material available at 10.1007/s00259-025-07456-z.

## Introduction

In positron emission tomography (PET), Gallium-68 (^68^Ga) has seen increased usage because of the commercial availability of ^68^Ge/^68^Ga generators in PET imaging. Moreover, novel ^68^Ga-labeled radiotracers, such as [^68^Ga]Ga-FAPI, will further expand the clinical use of ^68^Ga-PET imaging [1]. However, the large positron range of ^68^Ga decreases the image quality [2–7], depending on the underlying tissue density [2, 4, 5, 8–10]. Meanwhile, next-generation PET systems can achieve an intrinsic spatial resolutions comparable to the mean positron range of ^68^Ga [11–13], which was reported to be around ~ 2.2 mm in water [14]. Consequently, the large positron range of ^68^Ga is expected to have an even more pronounced impact on the image quality of these next-generation PET systems [15], making it essential to correct for this ^68^Ga-related resolution degrading effect.

Different positron range correction (PRC) techniques have been developed to address the positron range effect in ^68^Ga-PET imaging [16]. A simple approach, known as tissue-independent PRC, applies a uniform correction throughout the PET image by using the ^68^Ga positron range distribution in water as surrogate for soft tissue [14, 17–20]. However, this approach tends to overestimate the positron range effect in bone lesions while underestimating this effect for lung lesions, as shown in simulated phantom studies [18, 19]. To account for tissue-specific effects, tissue-dependent PRC approaches were developed considering additional tissues like bone and lung [8, 18–22]. For these approaches, the classification of the underlying tissue type was usually obtained through the segmentation of the computed tomography (CT) or magnetic resonance imaging (MRI) data that were acquired together with the PET data. A more advanced approach, known as tissue-dependent and spatially-variant PRC (TDSV PRC), accounts for both the underlying tissue composition and the boundaries between different tissue types, and is considered the most accurate PRC technique [8, 19, 21].

While different ^68^Ga-specific PRC techniques demonstrated benefits in the image quality of simulated data and phantom measurements [8, 14, 18–24], there are limited studies demonstrating the positive effect on the PET image quality of patient data. Previous studies reported that incorporating a tissue-independent PRC increased the contrast-to-noise ratio (CNR) and maximum standardized uptake value (SUV_max_) of the analyzed lesions compared to the non-PRC images [14, 17]. However, the effect of a TDSV PRC on the image quality of ^68^Ga-PET images has not yet been demonstrated for patient data.

In this study, a TDSV PRC technique for ^68^Ga-PET was developed. This technique was first validated by comparing the calculated positron range profiles with those generated through Monte Carlo simulations in patient data. Next, the improved quantification of artificially inserted lesions in five patients was evaluated. Finally, the effect of the TDSV PRC was investigated for clinical ^68^Ga-PET imaging of a cohort of 20 patients with bone, lung, and soft tissue lesions.

## Material and methods

### Patient data and acquisition protocol

This study included 20 patients (median weight: 82 kg, range: 53–102 kg) who underwent either [^68^Ga]Ga-PSMA (6/20) or [^68^Ga]Ga-DOTATOC (14/20) PET/CT imaging as part of their standard medical care between October 2022 and July 2023. Only inclusion criterion was the presence of at least one bone or lung lesion showing radiotracer uptake. The study received ethical approval (Date: 09-July-2021, No: 2021–2795) from the medical ethics committee METC azM/UM in Maastricht, The Netherlands, and informed consent was obtained from all patients prior to tracer administration. [^68^Ga]Ga-PSMA and [^68^Ga]Ga-DOTATOC were intravenously administered with an activity dosage of 1.5 MBq/kg. For patients receiving [^68^Ga]Ga-PSMA, an intravenous injection of furosemide (10 mg) was administered prior to tracer administration. Imaging was conducted 45 min post-injection, with a scanning duration of 3 min per bed position. For patients receiving [^68^Ga]Ga-DOTATOC, PET acquisition began 30 min post-injection with 4 min per bed position. Patients were instructed to void prior to the PET scan. Each patient was imaged using the Discovery MI LightBurst Digital 5-Ring Detector PET/CT system (GE HealthCare, United States). The PET scanner fulfilled the Fluorine-18 (^18^F) accreditation standards 1 and 2. Before the PET scan, a low-dose CT was carried out. The low-dose CT images were reconstructed using the ASIR-V algorithm using a blending level of 60%. The tube voltage was set at 120 kV and the automatic tube current modulation ranged from 10 to 60 mA. The noise index was 50%, the scanner’s rotation time was 0.5 s, while the pitch factor was 0.984.

### Tissue-dependent and spatially-variant positron range correction

Consistent with prior literature [8, 18–20, 22], this study employed three tissue types including lung, bone, and water with the latter as a surrogate for soft tissue (see Online Resource for details and rationale). The positron range distribution profiles of ^68^Ga in these three tissue types were obtained via Monte Carlo simulations in GATE 9.0 (Geant4 Application for Tomographic Emission, Geant4 10.6.2) [25, 26], with simulation details provided in the Online Resource. The profiles of those tissue types were mapped onto 3D homogeneous kernels. The kernel voxel size was set to match the voxel size (2.73 × 2.73 × 2.80 mm^3^) of the reconstructed PET data from the aforementioned PET/CT system. Finally, the kernel size was set to account for the maximum positron range in the corresponding tissue, resulting in 7 × 7 × 7 elements for water (8.8 mm), 25 × 25 × 25 for lung (32.3 mm), and 5 × 5 × 5 for bone (4.5 mm). Each kernel was normalized by dividing the voxel elements by the total number of annihilation events.

For each tissue type, a 3D binary tissue mask was produced following a voxel-based tissue classification based on the Hounsfield units (HU) of anatomical CT data. Specifically, the CT images were downsampled to match the respective PET voxel size. Then, each voxel was classified as water (HU: 3), lung (HU: -695), or bone (HU: 1226) using the nearest neighbor segmentation (see Online Resource for details on how the corresponding HU values were obtained for each tissue type). Subsequently, 3D binary masks were obtained for each tissue, where matching voxels were assigned a value of 1, or 0 otherwise.

During the image update of the PET reconstruction, the TDSV PRC was applied as follows: for each tissue type, the current image estimate was convolved with the corresponding tissue-specific positron range kernel, followed by an element-wise multiplication with the respective CT-based tissue-specific binary mask. Lastly, the PRC image estimate was obtained by summation of the three individual tissue-specific PRC image estimates, as described in Eq. (1):

1 $${\mathrm{reconImg}}_{\mathrm{PRC}}= \sum_{\text{i }=1}^{\mathrm{n}}\left(\left(\text{reconImg }\otimes {\mathrm{Kernel}}_{\mathrm{i}}\right) \odot {\mathrm{Mask}}_{\mathrm{i}}\right)$$

**Fig. 1:**
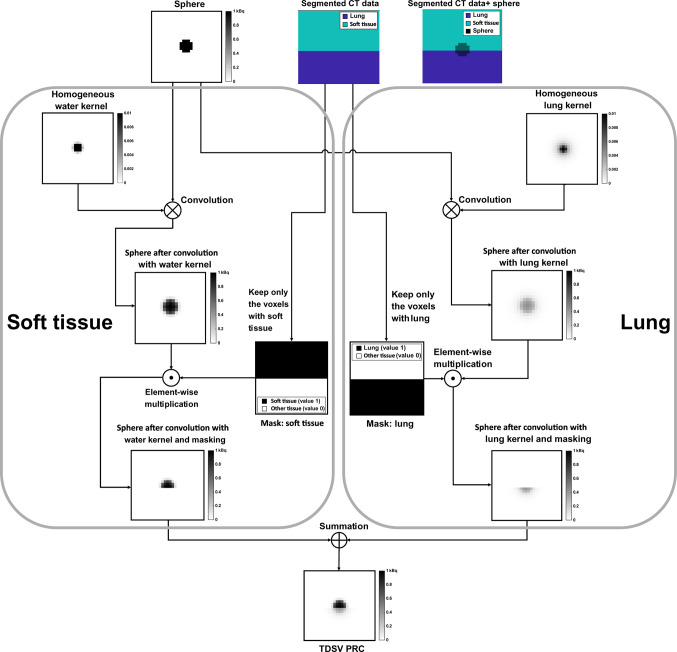
Application of TDSV PRC during the image update of iterative ^68^Ga-PET reconstruction. The ‘sphere’ represents the initial estimate. The ‘segmented CT data’ visualizes the distribution of the lung and soft tissue, while the ‘segmented CT data + sphere’ is used to demonstrate the overlap between the ‘sphere’ and the two tissues types

Where $\mathrm{n}$ is the number of the different tissues, which in this study is 3. The $\mathrm{reconImg}$ represents the PET image estimates during PET reconstruction, while ${\mathrm{Kernel}}_{\mathrm{i}}$ and ${\mathrm{Mask}}_{\mathrm{i}}$ are the kernel and the binary mask of tissue $\mathrm{i}$, respectively. The ${\mathrm{reconImg}}_{\mathrm{PRC}}$ is the PET image estimate during PET reconstruction after summing the individual tissue-specific PRC image estimates. To visualize this process, an illustration is provided in Fig. 1 in which for simplicity only two tissue types (lung and soft tissue) are used. Kertész et al. also proposed a similar TDSV PRC in a previous phantom study [8].

### PET reconstructions

All the PET image reconstructions were performed offline via the PET reconstruction toolbox Duetto (version 02.18), a Matlab-based research software package developed by GE HealthCare. The ordered subset expectation maximization (OSEM) reconstruction algorithm was employed with Time-of-Flight (ToF) information and Point-Spread-Function (PSF) modeling. Three different reconstructions were performed, without any PRC (non-PRC), using a tissue-independent PRC as previously published [14], and including the proposed TDSV PRC. For the PRC OSEM reconstructions, the respective PRC was applied in the forward and backward projection steps. All reconstructions employed voxel size of 2.73 × 2.73 × 2.80 mm^3^, while standard corrections were incorporated for radioactive decay, attenuation, scatter, dead time, randoms, and normalization. Reconstructions were performed using MATLAB R2023b (The MathWorks Inc., United States) on an Intel® Xeon® Gold 6130 CPU, implementing a multi-threaded approach with one thread per bed position.

To ensure fair comparison, the number of iterations, subsets, and post-reconstruction 3D Gaussian filtering were selected to achieve equivalent noise as measured by the standard deviation (SD) in liver and lung across the different reconstructions. Those parameters were selected in such a way that the recovery coefficient of the non-PRC images were compliant with the EARL 2 accreditation standard specified for ^18^F [27]. Specifically, all reconstructions were performed using 4 iterations, 34 subsets, and a post-reconstruction 3D Gaussian filter with a full-width-at-half-maximum of 6.0 mm (6.0 mm FWHM filter). This configuration achieved comparable SD values in both liver and lung regions. However, for the TDSV PRC reconstruction, a post-reconstruction 3D Gaussian filter with a 4.0 mm full-width-at-half-maximum (4.0 mm FWHM filter) was required to equalize the SD in lung regions, due to the application of a different positron range kernel in those areas.

### Performance evaluation of tissue-dependent and spatially-variant PRC

Initial evaluation was conducted using patient CT data to compare the positron range distribution profiles obtained from Monte Carlo simulations with the corresponding TDSV PRC profiles (see Online Resource). Further evaluation was performed via inserting artificial ^68^Ga-avid lesions in the patient PET data. Specifically, seven artificial homogeneous lesions, each with a radius of 5.0 mm, were inserted into the sinogram data of five patient datasets using an extension of Duetto toolbox. The corresponding binary masks used by the TDSV PRC were adjusted in those locations as well. The characteristics of these lesions are shown in Table 1. To avoid interference with other lesions, the artificial lesions were not placed near other lesions. Prior to lesion insertion, the positron range effects of the artificial lesions were obtained via Monte Carlo simulations (see Online Resource for details). Then, the activity of each lesion was adjusted to achieve the respective lesion-to-background activity ratio as shown in Table 1.

**Table 1:** Characteristics of the seven artificially created homogeneous lesions. The ‘Lesion name’ column lists the name of each artificial lesion as referenced in the study. The ‘Lesion activity ratio’ column shows the ratio of the lesion's activity compared to the background activity of the location where it was placed

Lesion name	Lesion type	Lesion location	Lesion activity ratio
Soft tissue_1_	Soft tissue	Inside liver (high uptake background)	3:1
Soft tissue_2_	Soft tissue	Inside other soft tissue (low uptake background)	16:1
Bone_1_	Bone	Inside bone tissue	16:1
Bone_2_	Soft tissue	Inside bone tissue	16:1
Lung_1_	Soft tissue	Inside the lung (completely surrounded by lung tissue)	16:1
Lung_2_	Soft tissue	Between lung and liver	10:1
Lung_3_	Soft tissue	Inside the lung (completely surrounded by lung tissue)	3:1

The artificial lesion characteristics were chosen to explore several different aspects. To examine the spill-out effect in different tissue configurations, the activity ratio of the corresponding artificial lesions was set at 16:1. To investigate the potential borderline detectability, the artificial lesion Lung_3_ was inserted with a 3:1 activity ratio in a low uptake background region. The artificial lesion Soft tissue_1_ also had a 3:1 ratio but was placed in a high uptake background region to investigate the spill-in effect from high background activity. For the artificial lesion Lung_2_, an intermediate activity ratio of 10:1 was selected, as it was placed between high and low uptake backgrounds.

For the five patients with artificial lesions, the reconstruction settings from the previous section were applied. For each artificial lesion, a spherical volume of interest (VOI) with a radius of 5.0 mm was created and placed at the known ground truth location using the software PMOD 3.7 (PMOD Technologies LLC, Switzerland). To investigate the effect of the TDSV PRC and the tissue-independent PRC, the percentage difference in SUV_max_, was calculated between the PRCs and non-PRC reconstructions for each artificial lesion. Additionally, the CNR was calculated per artificial lesion according to:

2 $$\mathrm{CNR}=\frac{{\mathrm{Mean}}_{\mathrm{Lesion}}-{\mathrm{Mean}}_{\mathrm{Background}}}{{\mathrm{SD}}_{\mathrm{Background}}}$$

With ${\mathrm{Mean}}_{\mathrm{Lesion}}$ referring to the measured mean activity concentration of the lesion, while ${\mathrm{SD}}_{\mathrm{Background}}$ and ${\mathrm{Mean}}_{\mathrm{Background}}$ denote the measured standard deviation and mean activity concentration of the surrounding background, respectively. The background activity concentration of each artificial lesion was measured by manually creating corresponding VOIs with a minimum volume of 10 mm^3^, positioned in close proximity to the lesion but far enough (distance > 5.0 mm) to minimize partial volume effects, as described in [17]. For the analysis, the percentage change in CNR of the PRCs against the non-PRC was used. Furthermore, the activity recovery for each artificial lesion per reconstruction was determined:

3 $$\text{Activity Recovery}=\frac{{\mathrm{Mean}}_{\mathrm{Lesion}}}{\text{True Activity}} \bullet 100 \%$$

With $\text{True Activity}$ describing the simulated activity of the corresponding artificial lesion as this was inserted into the patient data.

### Quantitative metrics for patient data

Lesions were delineated using the software PMOD after being identified by an experienced nuclear medicine physician (F.M.). A VOI was defined for each lesion using a 50% isocontour threshold based on the maximum voxel value. Additionally, lesions were categorized as soft tissue, bone, or lung lesions. Lesions featuring volumes greater than 10 cm^3^ were omitted as the potential benefit of a PRC for such lesions was demonstrated to be smaller than 5% [17]. From the remaining lesions, a maximum of the five largest lesions per tissue type per patient were selected to prevent potential bias. Additionally, for each lesion, the background activity concentration was quantified via corresponding VOIs, which were created in accordance with the methodology described in the previous section. All VOIs were generated in the reconstructions with TDSV PRC with 6.0 mm FWHM filter and, subsequently, mapped to the other corresponding reconstructions.

To compare the SD in liver across the different reconstructions while eliminating inhomogeneities in radiotracer uptake, the list-mode PET data for each patient were divided into two equal time segments per bed position. The first half covered the scan from start to midpoint, the second half from midpoint to end. Both segments were reconstructed separately, and the resulting images were subtracted to remove spatial uptake inhomogeneities. Then, a 20 mm radius spherical VOI was placed in the liver in such a way to avoid any overlap with lesions and the SD was measured on the subtracted image. The same method was also applied to measure the SD in the lung across the different reconstructions. This approach was similar to a previously described technique to eliminate inhomogeneous tracer uptake in whole-body dynamic PET imaging [28].

To evaluate lesion detectability and quantification, the SUV_max_ was obtained and the CNR was calculated for each lesion using Eq. (2). For bone and soft tissue lesions, the analysis was performed using a 6.0 mm FWHM filter across the different reconstructions. For lung lesions, this analysis was expanded to also include TDSV PRC images with 4.0 mm FWHM filter.

### Statistical analysis

Statistical analyses were performed for the SD in liver and lung regions, as well as for SUV_max_ and CNR, using R (version 4.3.2). For each metric, the percentage change was calculated comparing either PRC reconstructions to the non-PRC reconstructions. Additionally, for SUV_max_ and CNR, percentage changes between the TDSV PRC and tissue-independent PRC—both using a 6.0 mm FWHM filter—were computed. For lung lesions, percentage changes in SUV_max_ and CNR were calculated by comparing the TDSV PRC with a 4.0 mm FWHM filter to the non-PRC, tissue-independent PRC, and TDSV PRC with a 6.0 mm FWHM filter. All these percentage changes were tested for normality using the D'Agostino-Pearson omnibus normality test. The Wilcoxon signed-rank test was employed to assess statistical significance, as deviation from normal distribution was observed in some cases. The significance level was set at 0.05, and the p-value was adjusted for multiple comparisons using Bonferroni correction.

## Results

### Comparison of noise levels in liver and lung

**Fig. 2:**
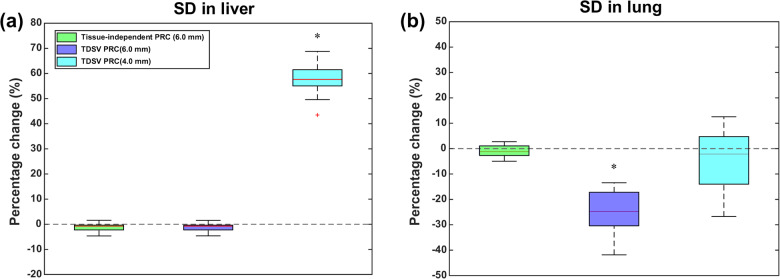
Percentage change (%) in SD of tissue-independent and TDSV PRC against the non-PRC images for liver (**a**) and lung (**b**). The asterisk (*) depicts a statistically significant difference (*p* < 0.05)

The percentage changes in noise (measured as SD) in liver and lung for TDSV and tissue-independent PRC compared to non-PRC are shown in Fig. 2. In liver, neither PRC method demonstrated statistically significant difference in the SD after applying a 6.0 mm FWHM filter (*p* = 0.06). However, in lung, the TDSV PRC with 6.0 mm FWHM filter significantly reduced the SD (-24.8%, *p* < 0.001), highlighting the necessity for a tissue-specific post-reconstruction filter for lung tissue, in contrast to the tissue-independent PRC (*p* = 0.52). When a 4.0 mm FWHM filter was used for the TDSV PRC, no significant difference was observed between TDSV PRC and non-PRC in the lung (*p* = 0.6), while the SD in the liver increased by 57.6% (*p* < 0.001). The median, minimum, and maximum SD values in liver and lung per reconstruction type are presented in Table S2 of the Online Resource.

### Artificial lesions

**Fig. 3:**
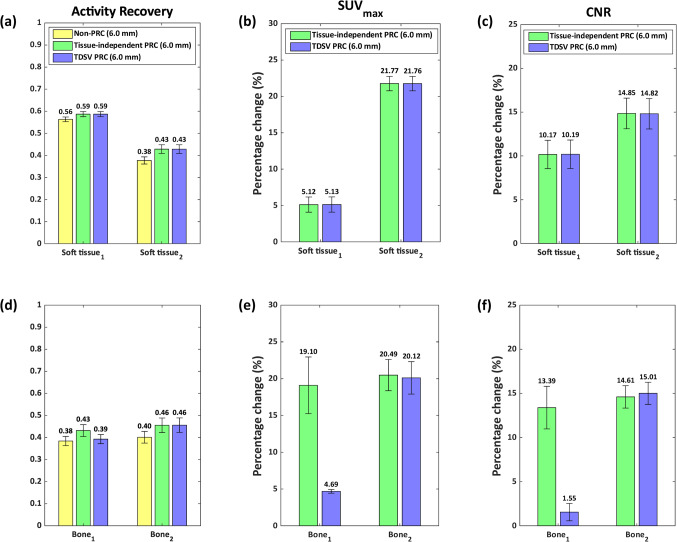
Quantification of artificial lesions. The left column presents the activity recovery, the middle column the percentage change (%) in SUV_max_ compared to non-PRC, and the right one the percentage change (%) in CNR compared to non-PRC for artificial lesions in soft tissue (**a**)-(**c**), and bone tissue (**d**)-(**f**). Each bar represents the mean ± standard error (SE), with the mean value annotated above

**Fig. 4:**
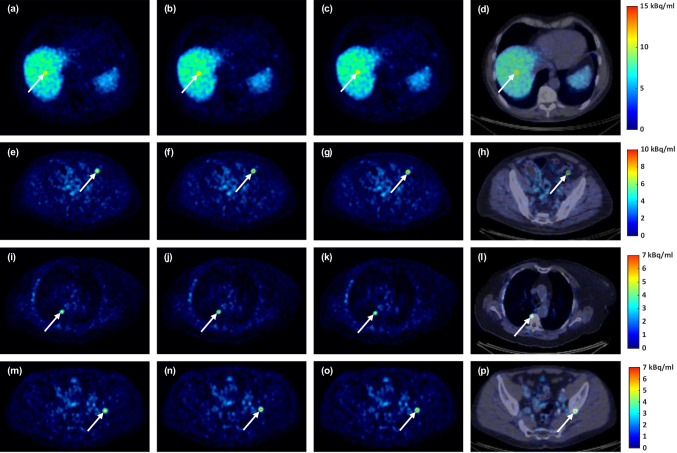
Image comparison of artificial soft tissue and bone lesions. From left to right, the columns show non-PRC, tissue-independent PRC, TDSV PRC, and fused PET/CT image for the TDSV PRC. Soft tissue_1_: (**a**)-(**d**). Soft tissue_2_: (**e**)-(**h**). Bone_1_: (**i**)-(**l**). Bone_2_: (**m**)-(**p**)

Figure 3 shows the activity recovery, the percentage change of the SUV_max_ in comparison to the non-PRC, and the percentage change of the CNR in comparison to the non-PRC for the artificial lesions in soft tissue and bone. For these type of lesions, only the results using a 6.0 mm FWHM filter are presented, as the 4.0 mm FWHM filter was shown to be suitable exclusively for lung tissue. In soft tissue, both PRC techniques had similar effects. In bone tissue, effects varied by the lesion type. Bone_1_ showed increased SUV_max_ and minor improvements in activity recovery and CNR with TDSV PRC, whereas tissue-independent PRC had larger effects. For Bone_2_, both PRCs had similar impacts. Figure 4 visually demonstrates the effect of the PRCs.

**Fig. 5:**
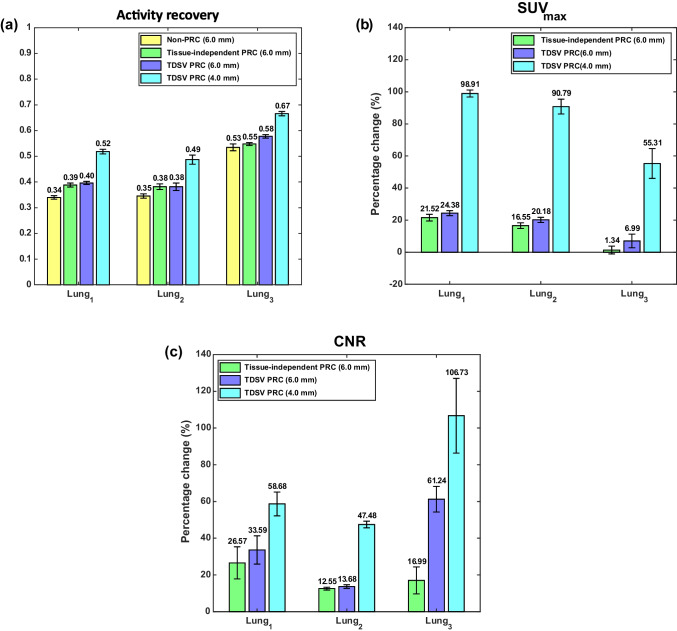
Artificial lesions in lung tissue: (**a**) activity recovery, (**b**) percentage change (%) in SUV_max_ compared to non-PRC, and (**c**) percentage change (%) in CNR compared to non-PRC. Each bar represents the mean ± standard error (SE), with the mean value annotated above

**Fig. 6:**
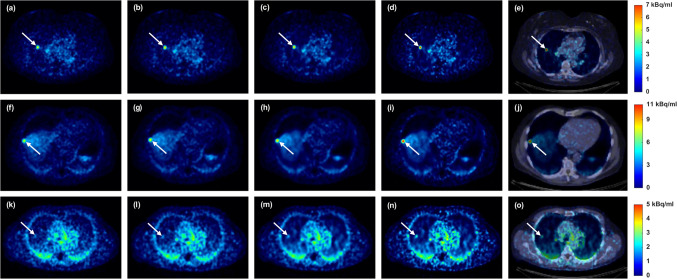
Image comparison of artificial lung lesions. From left to right, the columns show non-PRC, tissue-independent PRC, TDSV PRC with 6.0 mm FWHM filter, TDSV PRC with 4.0 mm FWHM filter, and PET/CT fusion for the TDSV PRC with 4.0 mm FWHM filter. Lung_1_: (**a**)-(**e**). Lung_2_: (**f**)-(**j**). Lung_3_: (**k**)-(**o**)

In Fig. 5, the PRC effects on the artificial lung lesions are shown. For the 6.0 mm FWHM filter, both PRCs improved activity recovery, SUV_max_, and CNR compared to non-PRC. In this case, although the 4.0 mm FWHM filter was shown to be suitable for lung tissue, results using the 6.0 mm FWHM filter are also presented to illustrate the differences in effect between the different reconstructions when applying the same filter. The TDSV PRC with 6.0 mm FWHM filter slightly outperformed the tissue-independent PRC, for Lung_1_ and Lung_2_, while greater increase was observed for Lung_3_. With a 4.0 mm FWHM filter, the TDSV PRC substantially increased all the metrics for the artificial lung lesions. Figure 6 visually demonstrates these effects, with Lung_3_ being sharper and notably more distinguishable using TDSV PRC.

### Quantitative analysis of lesions from patient data

In total, 93 lesions were identified, of which 24 were in [^68^Ga]Ga-PSMA and 69 in [^68^Ga]Ga-DOTATOC images. Of those lesions, 48 were categorized as soft tissue, 10 as lung, and the remaining 35 as bone. Similar to the artificial lesions, only the results obtained using a 6.0 mm FWHM filter are presented for bone and soft tissue lesions. For lung lesions, the 4.0 mm FWHM filter was found to be suitable for lung tissue; however, results with the 6.0 mm FWHM filter are also included to highlight the differences among reconstructions when applying the same post-reconstruction filter.

**Fig. 7:**
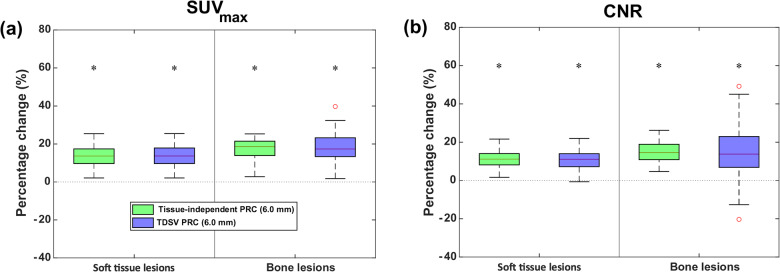
Percentage change (%) in (**a**) SUV_max_, and (**b**) CNR for TDSV or tissue-independent PRC vs. non-PRC in soft tissue and bone lesions. The asterisk (*) indicates significant difference (*p* < 0.001). No significant differences between the two PRCs (*p* = 1)

**Fig. 8:**
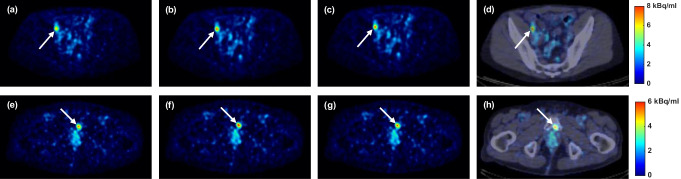
Image comparison of soft tissue and bone lesions. From left to right, the columns show non-PRC, tissue-independent PRC, TDSV PRC, and PET/CT fusion for the TDSV PRC. Soft tissue lesion (lymph node): (**a**)-(**d**). Bone lesion (metastasis): (**e**)-(**h**)

Both the tissue-independent and TDSV PRC significantly increased the SUV_max_ (13.6% vs. 13.7%, both *p* < 0.001) and the CNR (11.1% vs. 11.0%, both *p* < 0.001) for soft tissue lesions compared to non-PRC. For bone lesions, the tissue-independent PRC showed slightly higher increases (SUV_max_: 18.6% vs. 17.4%, both *p* < 0.001; CNR: 14.6% vs. 13.8%, both *p* < 0.001). These improvements are visible in Figs. 7 and 8. When comparing the TDSV PRC to the tissue-independent PRC, no statistically significant differences were observed for either lesion type (*p* = 1). Table S3 and Table S4 in the Online Resource present the median, minimum, and maximum SUV_max_ and CNR values per reconstruction type for soft tissue and bone lesions, respectively.

**Fig. 9:**
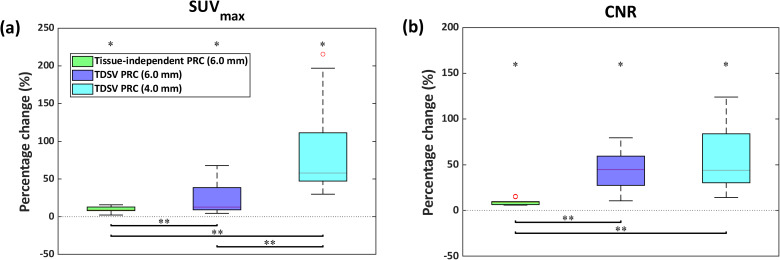
Percentage change (%) in (**a**) SUV_max_, and (**b**) CNR for TDSV or tissue-independent PRC vs. non-PRC in lung lesions. The asterisk (*) indicates a statistically significant difference to the non-PRC images (*p* < 0.05). The double asterisk (**) denotes statistically significant differences between the PRC images (*p* < 0.05)

**Fig. 10:**
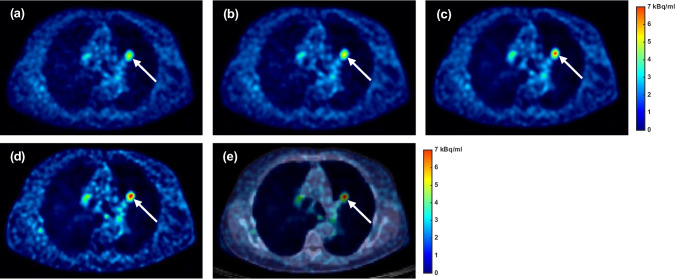
Image comparison for lung lesion. (**a**) Non-PRC. (**b**) Tissue-independent PRC. (**c**) TDSV PRC with 6.0 mm FWHM filter. (**d**) TDSV PRC with 4.0 mm FWHM filter. (**e**) PET/CT fusion for the TDSV PRC with 4.0 mm FWHM filter

As illustrated in Fig. 9, the tissue-independent PRC increased the SUV_max_ by 8.8% (*p* = 0.012) and CNR by 8.6% (*p* = 0.012) compared to non-PRC in lung lesions. The TDSV PRC with a 6.0 mm FWHM filter increased the SUV_max_ by 12.6% (*p* = 0.012) and CNR by 44.5% (*p* = 0.012), outperforming the tissue-independent PRC (SUV_max_: 4.1%, *p* = 0.012; CNR: 34.0%, *p* = 0.012). Using a 4.0 mm FWHM filter with the TDSV PRC increased the SUV_max_ (57.9%, *p* = 0.012) and the CNR (43.9%, *p* = 0.012) compared to non-PRC, and showed statistically significant differences from tissue-independent PRC (SUV_max_: 46.0%, *p* = 0.012; CNR: 32.5%, *p* = 0.012). While no difference in the CNR was observed between the two TDSV PRCs (*p* = 1), the SUV_max_ increased by 43.1% with the 4.0 mm FWHM filter (*p* = 0.012). These effects are visually evident in Fig. 10. Table S5 in the Online Resource reports the median, minimum, and maximum SUV_max_ and CNR values per reconstruction type for lung lesions.

## Discussion

In this study, a TDSV PRC for ^68^Ga was developed and evaluated on ^68^Ga-PET patient data. In addition to real lesions, simulated artificial lesions were also added to the patient data to evaluate this TDSV PRC. To the best of our knowledge, this is the first study exploring the potential benefits of a ^68^Ga-specific TDSV PRC method on patient data. Initial evaluation showed strong alignment between the profiles with only minor deviations between the profiles of TDSV PRC and full Monte Carlo simulations in CT sections obtained from patient data (see Online Resource). In artificial lesions, the TDSV PRC improved the activity recovery, SUV_max_, and CNR compared to non-PRC reconstructions. For real lesions, TDSV PRC with a 6.0 mm FWHM filter significantly increased SUV_max_ and CNR in soft tissue, bone, and lung lesions compared to the non-PRC. Due to the larger positron range kernel for lung tissue, a 4.0 mm FWHM filter was needed to match the non-PRC noise levels in the lung region, resulting in significant SUV_max_ and CNR increases for lung lesions.

Artificial lesions were introduced in five patients to validate the proposed TDSV PRC. As illustrated in Figs. 3 and 5, both tissue-independent and TDSV PRCs improved the activity recovery and increased the SUV_max_ and CNR compared to non-PRC reconstructions. As expected, for soft tissue lesions—where both techniques use the same positron range kernels—a similar effect was observed between tissue-independent and TDSV PRC. Notably, Soft tissue_1_ demonstrated better activity recovery than Soft tissue_2_ due to lower lesion-to-background activity ratio. Therefore, the spill-out from this lesion was partly compensated by spill-in from the surrounding environment. For lesions in bone tissue, the lesion tissue type influences the effect. Specifically, Bone_2_, which is a soft tissue lesion inside bone tissue, showed consistent incremental effect with both PRC methods. Bone_1_, a bone lesion inside bone tissue, displayed lower increments in metrics obtained from the TDSV PRC compared to the tissue-independent, likely due to the tendency of the latter one to overcorrect the positron range in bone tissue [18, 19]. Of the artificial lung lesions, Lung_3_ showed the highest activity recovery due to the relative high spill-in effects from the background region. The TDSV PRC with 6.0 mm FWHM filter outperformed the tissue-independent PRC for Lung_3_, as the latter underestimates the positron range in lung tissue [19]. For lung lesions with higher signal to background ratio, the TDSV PRC performed slightly better than tissue-independent PRC. With 4.0 mm FWHM filter, the TDSV PRC showed superior performance in all lung lesions. Moreover, TDSV PRC improved the detectability of Lung_3_, see Fig. 6, compared to the non-PRC and tissue-independent PRC.

Incorporating the TDSV PRC in the forward and backward projection steps significantly reduced the noise levels (measured as SD) in lung tissue. Studies that applied a PRC in a similar manner also reported reduced noise levels [8, 14, 17]. In this study, non-PRC images that complied with the EARL 2 accreditation standard for ^18^F served as the benchmark for noise levels. To achieve fair comparison between the TDSV PRC and the non-PRC reconstructions, the noise levels between them were equalized. By applying a 6.0 mm FWHM filter to all reconstructions, similar noise levels in the liver between PRC and non-PRC images was achieved, as shown in Fig. 2. This indicates that the 6.0 mm FWHM filter provided greater noise suppression in the liver than using a PRC alone. However, in lung tissue, a 4.0 mm FWHM filter was needed to equalized the noise levels between the TDSV PRC and the non-PRC reconstructions. Moreover, since the difference in noise levels between non-PRC and TDSV PRC becomes more pronounced as the positron range increases (see Fig. 2), adjustment is not expected to be necessary for bone tissue due to its small positron range. Consequently, applying a TDSV PRC in OSEM requires reconstructions with separate post-reconstruction filters for the lung and the rest of the body.

Both the TDSV and the tissue-independent PRC increased the SUV_max_ and the CNR compared to the non-PRC reconstructions across all patient lesions. For soft tissue lesions, both PRC methods demonstrated almost identical increment in comparison to the non-PRC reconstructions with no statistically significant difference between them. This observation was also confirmed in the artificial soft tissue lesions and it was expected, as the tissue-independent PRC applies a water-based kernel across the entire image, while the TDSV PRC utilizes the same kernel for soft tissue regions. For bone lesions, both PRC methods increased the SUV_max_ and CNR. The TDSV PRC demonstrated slightly lower SUV_max_ and CNR than the tissue-independent PRC, though the difference was not statistical significant. Furthermore, in the literature, the tissue-independent PRC was reported to result in overcorrections in bone tissue [18, 19], an observation also confirmed by the artificial lesions in the current study. An increment in the SUV_max_ and CNR was observed in the lung lesions after applying the TDSV PRC. In TDSV PRC, both metrics were also significantly larger than the tissue-independent PRC. This was expected as the tissue-independent PRC was reported to undercorrect the positron range effect in lung tissue [19]. Additionally, using a 4.0 mm FWHM filter with the TDSV PRC significantly increased the SUV_max_ compared to a 6.0 mm FWHM filter. Furthermore, as illustrated in Fig. 6, the TDSV PRC can potentially increase the detectability of small lung lesions that were borderline detectable compared to the non-PRC and tissue-independent PRC. In the literature, the implementation of a tissue-independent PRC in the Q.Clear reconstruction algorithm resulted in similar or better lesion detectability on patients compared to the non-PRC images, especially for small lesions [17]. This implies that more advanced PRC techniques, such as TDSV PRC, may enhance diagnostic outcomes for patients with lung lesions undergoing ^68^Ga-based radiotracers imaging.

The tissue-independent PRC increased the SUV_max_ and CNR (see Fig. 7) in the patient data, an observation that aligns with the findings in the literature [14, 17]. Increments in SUV_max_ and CNR for soft tissue, lung and bone lesions were reported from a larger patient cohort study employing the tissue-independent PRC [17]. However, direct comparison is hampered, as the previous study utilized a different reconstruction algorithm and reported the effect across lesions with different sizes.

A limitation of the proposed TDSV PRC is that it does not account for positron energy loss across different tissues, see Fig. S3 of the supplementary material. This leads to minor over- or under-estimations of the positron range. Overestimation occurs when the positrons were emitted in high-density tissues and travel to low-density ones, an observation also reported by Kertész et al. [8], while underestimation happens in the opposite situation. Furthermore, these estimation effects can vary in cases with complex tissue structures, especially when involving lung tissue, as shown in Fig. S3(d) of the Online Resource. However, these effects do not significantly impair the effectiveness of the proposed TDSV PRC.

An intrinsic challenge of the TDSV PRC is its dependency on the anatomical information obtained through the MRI or CT data. Issues in tissue segmentation may result in suboptimal or erroneous PRC that might result in artifacts. Advanced segmentation techniques, such as deep learning methods, have been developed to address potential segmentation issues in CT or MRI data [29, 30]. Additionally, patient motion can introduce artifacts, particularly respiratory motion in upper abdominal organs and basal lungs, causing misalignments between PET and CT or MRI data. These motion artifacts may account for the large range of values in the lung tissue observed in the TDSV PRC data, see Figs. 2 and 9. Incorporating motion correction techniques could potentially reduce these effects [31–33], which is crucial for the clinical implementation of the TDSV PRC.

In this study, lesion delineation was performed using a 50% threshold method without background correction. The VOIs were initially defined in the TDSV PRC reconstructions with a 6.0 mm FWHM filter and subsequently mapped to the other reconstructions. It should be noted that this method has limitations regarding delineation accuracy and does not reflect standard clinical practices. However, the primary aim of this study is to evaluate the impact of TDSV PRC on clinical ^68^Ga PET images, rather than to achieve optimal delineation accuracy. Furthermore, since TDSV PRC increases lesion SUV values, it directly affects both the delineated lesion volume and CNR, potentially complicating direct comparisons. To ensure a fair comparison of lesion quantification between TDSV PRC and images obtained from the other reconstructions, identical VOIs were applied to all cases.

Another challenge of the TDSV PRC is its computational cost. The proposed TDSV PRC increased the overall reconstruction time by 60.3% compared to non-PRC reconstructions. In contrast, the tissue-independent PRC increased the reconstruction time only by 2.8%. An increase in reconstruction time for the TDSV PRC has also been reported in previous studies [8, 19]. This indicates that further optimization is needed to reduce reconstruction time of the TDSV PRC to levels similar to those of non-PRC reconstructions.

Finally, a limitation of the lesion insertion tool is that it did not account for random and scatter effects. However, these effects are negligible due to the small size of the added lesions. Moreover, the primary focus of this study was to investigate the differences among non-PRC, tissue-independent PRC, and TDSV PRC techniques.

## Conclusion

This study demonstrated the beneficial effect of a TDSV PRC for ^68^Ga-PET image reconstruction. Artificial lesions added to patient data revealed that TDSV PRC enhanced the activity recovery, SUV_max_, and CNR of these lesions compared to non-PRC reconstructions. Furthermore, in a cohort of patient data, TDSV PRC increased the CNR and SUV_max_ across various lesion types, showing significant improvements over the non-PRC reconstructions. The most pronounced improvements were observed in lung lesions, highlighting this technique’s potential clinical benefit for patients with lung lesions.

## Supplementary Information

Below is the link to the electronic supplementary material.

Supplementary file1 (DOCX 337 KB)

## Data Availability

The datasets generated during and/or analysed during the current study are available from the corresponding author on reasonable request.
