# Supplementary material for: Clinical evaluation of tissue-dependent and spatially-variant positron range correction for Gallium-68 PET imaging
Source: Eur J Nucl Med Mol Imaging. 2025 Jul 28;53(2):1299–312. doi: 10.1007/s00259-025-07456-z (PMC12830501; doi:10.1007/s00259-025-07456-z)
Supplement: Supplementary file 1 — Supplementary file1 (DOCX 337 KB) [file 259_2025_7456_MOESM1_ESM.docx]

**Electronic Supplementary Material (ESM)**

**Title:** Clinical evaluation of tissue-dependent and spatially-variant positron range correction for Gallium-68 PET imaging

**Journal:** European Journal of Nuclear Medicine and Molecular Imaging

**Authors:** Prodromos Gavriilidis^1,2,3^, Michel Koole^3^, Felix M. Mottaghy^1,4^, Floris P. Jansen^5^, Roel Wierts^1,*^

**Affiliations:**

^1^ Department of Radiology and Nuclear Medicine, Maastricht University Medical Center, Maastricht, The Netherlands,

^2^ Research Institute for Oncology and Reproduction (GROW), Maastricht University, Maastricht, The Netherlands,

^3^ Nuclear Medicine and Molecular Imaging, Department of Imaging & Pathology, KU Leuven, Leuven, Belgium,

^4^ Department of Nuclear Medicine, RWTH Aachen University Hospital, Aachen, Germany,

^5^ Molecular Imaging, GE HealthCare, Waukesha, Wisconsin, USA,

^*^ Corresponding Author, e-mail address: roel.wierts@mumc.nl

**Tissue characterization phantom and positron range distribution profiles**

The Gammex 467 Tissue Characterization Phantom (Gammex Inc., United States) was used to determine relevant tissue types for the tissue-dependent and spatially-variant positron range correction (TDSV PRC). Computed tomography (CT) measurements were performed on that phantom using a Discovery MI LightBurst Digital 5-Ring Detector (GE HealthCare, United States) PET/CT system to map different tissues to their corresponding HU values. This phantom consists of a disk and a number of rod inserts. Each rod represents a different tissue type, shown in Table S1. The CT acquisition was performed using a tube voltage of 120 kVp and effective tube current of 152.4 mAs. To minimize potential artifacts, the high-density tissues were evenly distributed throughout the phantom, see Fig. S1.

**Table S1:** The different tissues with their corresponding density (ρ) in g/cm^3^, electron density relative to water (ρ_e_^w^) and elemental composition. The elements are the Hydrogen (H), Oxygen (O), Carbon (C), Nitrogen (N), Chlorine (Cl), Calcium (Ca), Phosphorus (P), and Magnesium (Mg).

|  |  |  | **Elemental composition (%)** | | | | | | | |
| --- | --- | --- | --- | --- | --- | --- | --- | --- | --- | --- |
| **Tissues** | **ρ** | **ρ_e_^w^** | **H** | **O** | **C** | **N** | **Cl** | **Ca** | **P** | **Mg** |
| Cortical bone | 1.82 | 1.69 | 2.66% | 39.08% | 30.34% | 0.99% | 0.04% | 26.48% |  | 0.41% |
| CB2 - 50% | 1.56 | 1.45 | 4.03% | 34.13% | 40.34% | 1.52% | 0.07% | 19.62% |  | 0.30% |
| CB2 - 30% | 1.33 | 1.26 | 5.60% | 28.47% | 51.77% | 2.12% | 0.09% | 11.77% |  | 0.18% |
| B-200 bone | 1.15 | 1.10 | 6.94% | 19.82% | 56.62% | 1.81% | 0.11% | 6.39% | 8.31% |  |
| Inner bone | 1.14 | 1.09 | 6.97% | 19.71% | 56.73% | 1.79% | 0.11% | 6.38% | 8.30% |  |
| Liver | 1.09 | 1.07 | 8.40% | 18.50% | 68.41% | 2.25% | 0.14% | 2.26% |  | 0.03% |
| Brain | 1.05 | 1.04 | 9.77% | 16.34% | 72.13% | 1.69% | 0.07% | 0.00% |  |  |
| Muscle | 1.05 | 1.02 | 8.39% | 18.45% | 68.50% | 2.22% | 0.14% | 2.26% |  | 0.03% |
| Solid water | 1.02 | 0.99 | 8.39% | 18.40% | 68.59% | 2.19% | 0.14% | 2.26% |  | 0.03% |
| Breast | 0.98 | 0.96 | 8.97% | 16.45% | 71.36% | 2.14% | 0.13% | 0.93% |  | 0.01% |
| Adipose | 0.95 | 0.94 | 9.44% | 14.86% | 73.50% | 2.07% | 0.13% | 0.00% |  |  |
| LN-450 lung | 0.45 | 0.43 | 7.44% | 20.69% | 58.03% | 1.97% | 0.08% |  |  | 11.22% |
| LN-300 lung | 0.29 | 0.28 | 7.43% | 20.71% | 57.86% | 1.96% | 0.08% |  |  | 11.19% |

**Fig. S1:** Configuration of tissue characterization phantom.

Monte Carlo simulations in GATE 9.0 (Geant4 Application for Tomographic Emission, Geant4 10.6.2) were performed to obtain the positron range distribution profiles of ^68^Ga for each tissue type in Table S1 [1, 2]. A 20 × 20 × 20 cm^3^ uniform cube of the corresponding tissue was defined and at the center a ^68^Ga positron emitting point source of 1 µm diameter was placed. Positron annihilation, electron ionization, multiple scattering and bremsstrahlung were taken into account during the simulations. Each simulation generated about 17.8 million annihilation events, with emission and annihilation coordinates used to create the positron range distribution profiles.

The positron range distribution profiles of the different tissues, are depicted in Fig. S2. The solid water and the different soft tissues exhibit notably similar shapes and positron range. This similarity suggests that solid water serves as an appropriate surrogate for soft tissues when considering positron range effects. For bone or bone-like tissues, there are differences in shape and positron range, see Fig. S2(b). While solid water, B-200 bone, and inner bone exhibit relatively minor differences, higher-density bone tissues demonstrate more pronounced differences, with cortical bone showing the largest difference. For lung tissues, LN-300 lung exhibited the most pronounced difference in shape and positron range compared to solid water, see Fig. S2(c).

**Fig. S2:** Positron range distribution profiles of the different tissues from the Gammex 467 Tissue Characterization Phantom. **(a)** Solid water and soft tissues. **(b)** Solid water alongside bone or bone-like tissues. **(c)** Solid water and lung-like tissues.

Based on the positron range profiles presented in Fig. S2, the tissues selected for this study were solid water (water), LN-300 lung (lung), and cortical bone (bone). LN-300 lung and cortical bone were found to be a suitable representations for lung and bone tissues, respectively, as they showed the greatest deviations in their positron range profiles from water. Additionally, if a water kernel is applied, those two selected tissues are prone to error due to greater overestimation (for cortical bone) or underestimation (for LN-300 lung), as they exhibit large differences in their positron range profiles compared to water. Moreover, the Hounsfield Unit (HU) values of the selected tissues were consistent with those found in the current patient data. This confirms the inclusion of bone, soft tissue, and lung, aligning with existing literature [3-8].

The profiles of the three selected tissues were mapped onto 3D homogeneous kernels. The kernel voxel size was set to match the PET/CT system (2.73 x 2.73 x 2.80 mm^3^). The kernel size was selected to account for the maximum positron range in the corresponding tissue. This resulted in a kernel size of 7 × 7 × 7 elements for solid water (maximum positron range: 8.8 mm), 25 x 25 x 25 for LN-300 lung (maximum positron range: 32.3 mm), and 5 × 5 × 5 for cortical bone (maximum positron range: 4.5 mm). Each kernel was normalized by dividing each voxel element by the total number of annihilation events.

**Comparison of positron range distribution profiles from patient CT data**

Four cubic sections of 68.25 x 68.25 x 70.00 mm^3^ containing tissue boundaries were obtained from the patient CT data. Each section was voxelated and each voxel was classified as lung (HU: -695), water (HU: 3), or bone (HU: 1226) using the nearest neighbor segmentation. Monte Carlo simulations of ^68^Ga point sources were then performed to obtain the positron range distribution profiles, using same simulation configurations as described in the previous section of the Online Resource. In two simulations, the point source was placed inside tissue with higher density than the boundary tissue, while in the other two simulations, it was placed inside tissue with lower density. The resulting profiles were compared with the TDSV PRC profiles based on the shape and the area under the curve (AUC).

Figure S3 displays the four patient CT data sections along with their corresponding distribution profiles from Monte Carlo simulations and TDSV PRC. Overall, the TDSV PRC profiles showed substantial overlap with the Monte Carlo simulations, with minor discrepancies in the AUC. In Fig. S3(a), the AUC of the TDSV PRC was 6.6% lower than that of the Monte Carlo profile, while in Fig. S3(b), it was 3.5% higher. Fig. S3(c) showed a 1.1% overestimation of the AUC from the TDSV PRC compared to Monte Carlo. Notably, in Fig. S3(d), the AUC of the TDSV PRC profile was 11.9% lower compared to the corresponding profile from Monte Carlo simulations. This underestimation is attributed to the greater complexity of the tissue structure in that specific section.

**Fig. S3:** Cross-sections of labeled CT data (left) with corresponding distribution profiles derived from Monte Carlo simulations and the TDSV PRC (right). **(a)** Point source within soft tissue adjacent to bone. **(b)** Point source within bone next to soft tissue. **(c)** Point source in soft tissue bordering the lung. **(d)** Point source located in the lung adjacent to soft tissue. Red dashed lines in the distribution profiles indicate the interfaces where tissue composition changes, while the black arrow shows the direction of the profile. A.U. stands for arbitrary unit.

**Monte Carlo simulations to determine the positron range effect of artificial lesions**

Before inserting the artificial lesions into the sinogram, their corresponding positron range effect needs to be obtained. This was achieved utilizing Monte Carlo simulations. Specifically, a 20 × 20 × 20 cm³ cube was defined, with tissue properties based on the location of the respective artificial lesion described in Table 1 of the manuscript. At the center of this cube, a homogeneous sphere with a radius of 5.0 mm was created, with tissue properties corresponding to the appropriate artificial lesion type presented in Table 1 of the manuscript. Within this sphere, a spherical ^68^Ga source with 5.0 mm radius was placed. All other simulation configurations were as described in the first section of the Online Resource.

**Quantitative metrics per reconstruction type**

**Table S2:** Summary of noise in the liver and lung as measured by the standard deviation (SD), reported as median (minimum – maximum).

| **Reconstructions** | **Liver** | **Lung** |
| --- | --- | --- |
| Non-PRC (6.0 mm) | 1.10 (0.76 - 1.92) | 0.18 (0.11 - 0.34) |
| Tissue-independent PRC (6.0 mm) | 1.09 (0.77 - 1.91) | 0.18 (0.11 - 0.34) |
| TDSV PRC (6.0 mm) | 1.09 (0.77 - 1.91) | 0.14 (0.07 - 0.28) |
| TDSV PRC (4.0 mm) | 1.71 (1.21 - 3.01) | 0.17 (0.09 - 0.35) |

**Table S3:** Summary of maximum standardized uptake value (SUV_max_) and contrast-to-noise ratio (CNR) for soft tissue lesions, presented as median (minimum–maximum).

| **Reconstructions** | **SUV_max_** | **CNR** |
| --- | --- | --- |
| Non-PRC (6.0 mm) | 11.7 (3.8 – 30.9) | 11.1 (1.4 – 57.0) |
| Tissue-independent PRC (6.0 mm) | 13.1 (4.7 – 31.8) | 12.3 (1.6 – 64.6) |
| TDSV PRC (6.0 mm) | 13.0 (4.7 – 31.8) | 12.4 (1.6 – 64.6) |

**Table S4:** Summary of SUV_max_ and CNR for bone lesions, provided as median (minimum–maximum).

| **Reconstructions** | **SUV_max_** | **CNR** |
| --- | --- | --- |
| Non-PRC (6.0 mm) | 6.7 (1.9 – 39.4) | 19.8 (4.1 – 203.1) |
| Tissue-independent PRC (6.0 mm) | 7.9 (2.1 – 44.5) | 22.7 (4.6 – 227.8) |
| TDSV PRC (6.0 mm) | 7.5 (2.1 – 44.6) | 22.4 (4.2 – 177.4) |

**Table S5:** Summary of SUV_max_ and CNR for lung lesions, expressed as median (minimum–maximum).

| **Reconstructions** | **SUV_max_** | **CNR** |
| --- | --- | --- |
| Non-PRC (6.0 mm) | 2.7 (1.4 – 4.1) | 7.8 (2.3 – 20.1) |
| Tissue-independent PRC (6.0 mm) | 3.0 (1.4 – 4.4) | 8.5 (2.4 – 23.2) |
| TDSV PRC (6.0 mm) | 3.4 (1.5 – 4.8) | 11.9 (3.3 – 32.1) |
| TDSV PRC (4.0 mm) | 5.0 (1.8 – 10.5) | 12.1 (3.4 – 37.0) |

**References**

1. Jan S, Santin G, Strul D, Staelens S, Assié K, Autret D, et al. GATE: a simulation toolkit for PET and SPECT. Phys Med Biol. 2004;49:4543. <https://doi.org/10.1088/0031-9155/49/19/007>.
2. Jan S, Benoit D, Becheva E, Carlier T, Cassol F, Descourt P, et al. GATE V6: a major enhancement of the GATE simulation platform enabling modelling of CT and radiotherapy. Phys Med Biol. 2011;56:881. <https://doi.org/10.1088/0031-9155/56/4/001>.
3. Cal-González J, Herraiz JL, España S, Vicente E, Herranz E, Desco M, et al. Study of CT-based positron range correction in high resolution 3D PET imaging. Nucl Instrum Methods Phys Res A. 2011;648:S172-S5. <https://doi.org/10.1016/j.nima.2010.12.041>.
4. Cal-González J, Pérez-Liva M, Herraiz JL, Vaquero JJ, Desco M, Udías JM. Tissue-Dependent and Spatially-Variant Positron Range Correction in 3D PET. IEEE Trans Med Imaging. 2015;34:2394-403. <https://doi.org/10.1109/TMI.2015.2436711>.
5. Bertolli O, Eleftheriou A, Cecchetti M, Camarlinghi N, Belcari N, Tsoumpas C. PET iterative reconstruction incorporating an efficient positron range correction method. Physica Medica. 2016;32:323-30. <https://doi.org/10.1016/j.ejmp.2015.11.005>.
6. Rukiah AL, Meikle SR, Gillam JE, Kench PL. An investigation of 68Ga positron range correction through de-blurring: A simulation study. 2018 IEEE Nuclear Science Symposium and Medical Imaging Conference Proceedings (NSS/MIC). Sydney, NSW, Australia: IEEE; 2018. p. 1-2. <https://doi.org/10.1109/NSSMIC.2018.8824301>.
7. Kertész H, Beyer T, Panin V, Jentzen W, Cal-Gonzalez J, Berger A, et al. Implementation of a Spatially-Variant and Tissue-Dependent Positron Range Correction for PET/CT Imaging. Front Physiol. 2022;13. <https://doi.org/10.3389/fphys.2022.818463>.
8. Li C, Scheins J, Tellmann L, Issa A, Wei L, Shah NJ, Lerche C. Fast 3D kernel computation method for positron range correction in PET. Phys Med Biol. 2023;68:025004. <https://doi.org/10.1088/1361-6560/acaa84>.
